# Optochemical control of RNA interference in mammalian cells

**DOI:** 10.1093/nar/gkt806

**Published:** 2013-09-10

**Authors:** Jeane M. Govan, Douglas D. Young, Hrvoje Lusic, Qingyang Liu, Mark O. Lively, Alexander Deiters

**Affiliations:** ^1^Department of Chemistry, North Carolina State University, Raleigh, NC 27695, USA, ^2^Department of Chemistry, College of William & Mary, Williamsburg, VA 32187, USA, ^3^Center for Structural Biology, Wake Forest University School of Medicine, Winston-Salem, NC 27157, USA and ^4^Department of Chemistry, University of Pittsburgh, Pittsburgh, PA 15260, USA

## Abstract

Short interfering RNAs (siRNAs) and microRNAs (miRNAs) have been widely used in mammalian tissue culture and model organisms to selectively silence genes of interest. One limitation of this technology is the lack of precise external control over the gene-silencing event. The use of photocleavable protecting groups installed on nucleobases is a promising strategy to circumvent this limitation, providing high spatial and temporal control over siRNA or miRNA activation. Here, we have designed, synthesized and site-specifically incorporated new photocaged guanosine and uridine RNA phosphoramidites into short RNA duplexes. We demonstrated the applicability of these photocaged siRNAs in the light-regulation of the expression of an exogenous green fluorescent protein reporter gene and an endogenous target gene, the mitosis motor protein, Eg5. Two different approaches were investigated with the caged RNA molecules: the light-regulation of catalytic RNA cleavage by RISC and the light-regulation of seed region recognition. The ability to regulate both functions with light enables the application of this optochemical methodology to a wide range of small regulatory RNA molecules.

## INTRODUCTION

Light irradiation is an excellent external trigger to afford high-resolution control over biological processes, as irradiation can be precisely controlled in time, space and amplitude. One approach to regulate the activity of a biologically functional molecule with light is through the installation of a photocleavable protecting group onto the molecule, thereby rendering it inactive—a process that has been termed ‘caging’ (1–8). A brief irradiation with non-damaging UV light removes the caging group and restores activity of the biomolecule. This methodology has been successfully applied to the light-regulation of gene expression through caged antisense agents (9–14), caged mRNA (15), caged DNA decoys (16), caged triplex-forming oligonucleotides (17), caged proteins (18–20), caged small molecules (21,22) and recently to caged miRNA antagomirs (23,24).

Short interfering RNAs (siRNAs) are powerful gene-silencing tools that have been widely applied to the study of gene function and gene regulation (25,26). Consisting 19–21 nucleotides, siRNAs are double-stranded RNAs that are processed through the RNA interference pathway to inhibit gene expression in a sequence-specific manner (27). siRNAs are typically either transfected or injected into cells, and on entrance into RISC, the 5′ phosphate of the antisense strand is bound within the PIWI domain of argonaute (28). The catalytic domain of argonaute subsequently cleaves the sense strand, which is then removed from RISC. Structural studies have shown that the anchored antisense strand remains in a helical conformation within RISC (29). This exposes the seed region, nucleotides 2–8, to site-specifically bind to the mRNA target, providing the basis for initial target site recognition. siRNAs, in contrast to miRNAs, are completely complementary to the mRNA target, which is cleaved at nucleotides 10–11 of the antisense strand by the catalytic subunit of argonaute, followed by further degradation outside of RISC (30). Through kinetic studies of RISC, it was concluded that the A-form helix of the siRNA:mRNA duplex in RISC is essential for siRNA activity (31). The replacement of an essential nucleobase with a mismatched nucleotide into the antisense strand creates a ‘bulge’ within the siRNA:mRNA duplex and can render the siRNA completely inactive owing to the structural distortion of the A-form (32). Consequently, the maintenance of this structural motif for mRNA recognition is a viable target for a caging strategy.

Recently, different caging approaches have been applied to the optochemical regulation of siRNA function. For example, Friedman *et al.* incorporated sterically demanding cyclo-dodecyl-4,5-dimethoxy-2-nitrophenylethyl groups at each terminus of the siRNA (33), thus rendering the siRNA molecule inactive until UV irradiation restored 80% of gene-silencing activity. The 4,5-dimethoxy-2-nitrophenylethyl (DMNPE) caging group was incorporated non-specifically throughout 2′ fluoro-siRNA by Monroe *et al.* to achieve optochemical control over siRNA activity (34). The DMNPE-caged 2′ fluoro-siRNAs did not show full inhibition of siRNA function, and siRNA activity could not be completely restored through UV irradiation. However, functionality in a zebrafish model system was demonstrated. In a third approach, Heckel *et al.* incorporated a 2-(2-nitrophenyl)propyl (NPP)-caged deoxyguanosine nucleotide into the antisense strand of an siRNA (35). By NPP-caging one of the nucleotides at position 9–11, siRNA activity was reduced to 10%. However, some instability of the caging group was observed, as increased RNAi activity was apparent after 28 h in cells that were kept in the dark.

**Scheme 1.**
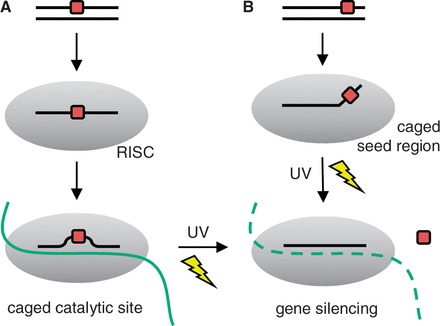
Two siRNA light-activation approaches. Caged nucleotides are positioned (**A**) near the cleavage site or (**B**) at the seed region of the siRNA agent, leading to gene expression by preventing RISC cleavage or mRNA target recognition. On UV irradiation, the caging groups are cleaved resulting in the silencing of gene expression through RNA interference.

Here, we report the first synthesis of 6-nitropiperonyloxymethyl (NPOM)-caged guanosine and uridine phosphoramidites and their site-specific incorporation into siRNA reagents that are composed of ribonucleic acids at the caging sites. The caged siRNA is expected to be functionally inactive and not silence gene expression until a brief UV exposure removes the caging groups and activates the siRNA reagent resulting in gene silencing (Scheme 1). We initially tested the light-activation of siRNAs with a green fluorescent protein (GFP) reporter as a proof-of-concept, but subsequently demonstrated the light-activation of endogenous Eg5 silencing. Eg5 is a mitosis motor protein involved in spindle formation and movement in cell division (36–38). The inhibition of Eg5 leads to a binucleated cell phenotype (39). This, for the first time, demonstrated the applicability of light-activated RNAi in the silencing of an endogenous gene via a photocaged RNA nucleobase strategy.

Multiple sites within an siRNA reagent are conceivable targets for the installation of caging groups to optochemically regulate its function. We selected two different sites for the introduction of our NPOM-caged nucleotides: (i) within the seed region of the siRNA (nucleotides 2–8, Scheme 1B) and (ii) surrounding the argonaute cleavage site of the siRNA bound to RISC (nucleotides 9–11, Scheme 1A) (31). The seed region of the small non-coding RNA is an important motif found in both siRNAs and miRNAs, guiding the reagents to their target mRNAs. However, a major difference between both classes of gene-silencing agents is that miRNAs do not cleave their target mRNA due to the typical incomplete sequence complementarity between the miRNA and its target, but rather suppresses the translation of the target gene (40). In contrast, RNA cleavage by argonaute commonly occurs in the case of perfect homology between siRNA and mRNA target sequences (41,42). For this reason, the introduction of caging groups within the seed region is an attractive tool not only for the light-regulation of siRNAs but also of miRNAs. Based on the prevalence of guanosine and uridine nucleotides within the seed region (nucleotides 2–8) and at the argonaute cleavage site (nucleotides 9–11) within the selected siRNA reagents, novel NPOM-caged guanosine and NPOM-caged uridine phosphoramidites were synthesized, as discussed later in the text. The availability of both NPOM-caged guanosine and NPOM-caged uridine building blocks affords the needed flexibility for sequence selection for caging group installation. The site-specific installation of NPOM-caged nucleotides at specific positions allowed us to confirm that base-pairing at these nucleotides with the mRNA target is essential for siRNA activity and therefore represents a general approach to the generation of light-activated siRNAs.

## MATERIALS AND METHODS

### RNA synthesis protocol

GFP siRNA was purchased from Dharmacon. Non-caged and caged RNA synthesis was performed using an Applied Biosystems 394 Automated DNA/RNA Synthesizer (Foster City, CA, USA) and standard β-cyanoethyl phosphoramidite chemistry. The caged siRNAs were synthesized using 40-nmole scale, low volume solid phase supports obtained from Glen Research (Sterling, VA, USA). Reagents for automated RNA synthesis were also obtained from Glen Research. Standard synthesis cycles provided by Applied Biosystems were used for all normal bases using 2 min coupling times. The coupling time was increased to 10 min for the positions at which the caged-U- and caged-G-modified phosphoramidites were incorporated. Each synthesis cycle was monitored by following the release of dimethoxytrityl (DMT) cations after each deprotection step. No significant loss of DMT was noted following the addition of the caged-U or caged-G to the RNA, so 10 min was sufficient to allow maximal coupling of the caged nucleotides. Yields of caged RNAs were close to theoretical values routinely obtained.

### Melting temperatures

The melting temperature (T_m_) of each siRNA was measured using a Cary 100 Bio UV/Vis spectrometer with a temperature controller (Varian). The two complementary strands of siRNAs (1 µM) were incubated in 0.15 M NaCl, 0.05 M NaH_2_PO_4_, pH 7.2, buffer. The samples were protected from light or irradiated at 365 nm with a UV transilluminator (3 mW cm^−^^2^) for 20 min, heated to 100°C for 2 min, and then cooled to 20°C at a rate of 2°C/min, held at 20°C for 5 min and then heated to 100°C at a rate of 2°C/min. Absorbance was recorded at 260 nm every 1°C. The T_m_ was determined by the maximum of the first derivative of the absorbance versus temperature plot. Standard deviations were calculated from three individual experiments.

### Flow cytometry analysis

Human embryonic kidney (HEK) 293T cells were grown at 37°C, 5% CO_2_ in Dulbecco’s modified Eagle’s medium (DMEM; Hyclone), supplemented with 10% fetal bovine serum (Hyclone) and 10% streptomycin/penicillin (MP Biomedicals). Cells were passaged into 24-well plates (1 ml per well, ∼4 × 10^4^ cells per well) and grown to ∼70% confluence within 24 h. The medium was changed to OPTIMEM (Invitrogen), and the cells were transfected with 0.5 µg pEGFP-N1 (Clontech) and 0.5 µg pDsRed-N1 monomer (Clontech) and 40 pmol siRNAs with X-tremeGENE siRNA reagent (3:2 reagent/RNA ratio, Roche). All transfections were performed in triplicate. Cells were incubated at 37°C for 4 h, the transfection medium was removed and 1 ml phosphate buffered saline (PBS, pH 7.4) was added. The cells were irradiated for 5 min on a UV transilluminator (365 nm, 3 mW cm^−^^2^). PBS was removed and DMEM media was added, followed by a 48-h incubation at 37°C, 5% CO_2_, and the cells (20 000) were assayed for fluorescence by flow cytometry. Analysis was performed on a FACSCalibur (Becton-Dickinson) instrument, using a 488-nm excitation laser with a 530-nm band pass filter (GFP) and a 633-nm excitation argon laser and 661-nm band pass filter (DsRed). Fluorescence was analyzed using the Cellquest Pro Software. For each of the triplicates, the data were averaged, and standard deviations were calculated.

### Phenotypic Eg5 inhibition assay

HeLa cells were grown at 37°C, 5% CO_2_ in DMEM (Hyclone), supplemented with 10% fetal bovine serum (Hyclone) and 10% streptomycin/penicillin (MP Biomedicals). Cells were passaged into 4-well chamber slide (1 ml/well, ∼4 × 10^4^ cells/well) and grown to ∼70% confluence within 24 h. The medium was changed to OPTIMEM (Invitrogen) and the cells were transfected with 40 pmol siRNAs with X-tremeGENE siRNA reagent (3:2 reagent/RNA ratio, Roche). Cells were incubated at 37°C for 4 h, the transfection medium was removed and 0.25 ml PBS (pH 7.4) was added. The cells were irradiated for 5 min on a UV transilluminator (365 nm, 3 mW cm^−^^2^). DMEM media was added, and the cells were incubated at 37°C, 5% CO_2_ for 48 h. The cells were fixed with formaldehyde (3.75%) and permeabilized with Triton-100x, strained with Alexa Fluor 488 Phalloidin (ex/em 488 nm/495–630 nm, Invitrogen) and 4′,6-diamidino-2-phenylindole (DAPI) (ex/em 405 nm/410–495 nm, Invitrogen). Cells were imaged on a Zeiss LSM 710 confocal microscope (40× oil objective).

### Quantitative real time-polymerase chain reaction

HeLa cells were passaged into six-well plates (2 ml per well, ∼2 × 10^5^ cells per well) and grown to ∼70% confluence within 24 h. The medium was changed to OPTIMEM (Invitrogen), and the cells were transfected with 40 pmol siRNAs with X-tremeGENE siRNA reagent (3:2 reagent/RNA ratio, Roche). All transfections were performed in triplicate. Cells were incubated at 37°C for 4 h, the transfection medium was removed and 1 mL PBS (pH 7.4) was added. The cells were irradiated for 5 min on a UV transilluminator (365 nm, 3 mW cm^−^^2^). DMEM media was added, and the cells were incubated at 37°C, 5% CO_2_ for 48 h. RNA was isolated with TRIZOL reagent (Invitrogen). cDNAs were synthesized with Superscript Reverse Transcriptase II (Invitrogen) and quantitative real time-polymerase chain reactions (RT-PCRs) were performed with Eg5 forward primer 5′ CAGCTGAAAAGGAAACAGCC, Eg5 reverse primer 5′ ATGAACAATCCACACCAGCA (37), GAPDH forward primer 5′ TGCACCACCAACTGCTTAGC and GAPDH reverse primer 5′ GGCATGGACTGTGGTCATGAG (43). The threshold cycles of each sample were normalized to the GAPDH housekeeping gene, and the inhibition of gene silencing is represented as percentage of Eg5 expression. For each of the triplicates, the data were averaged, and standard deviations were calculated.

## RESULTS

### Synthesis of NPOM-caged guanosine and uridine phosphoramidites

**Scheme 2.**
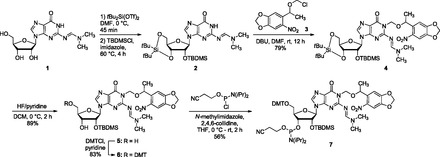
Synthesis of the NPOM-caged guanosine phosphoramidite **7** from commercially available **1**. Dimethylformamide (DMF), *tert*-butyldimethylsilyl chloride (TBDMSCl), dichloromethane (DCM), 1,8-diazabicyclo[5.4.0]undec-7-ene (DBU), di(*p*-methoxyphenyl)phenyl-methyl chloride (DMTCl) and tetrahydrofuran (THF).

Caged guanosine and uridine RNA phosphoramidites were prepared to synthesize caged RNA oligonucleotides and to site-specifically incorporate caged nucleotides into siRNA reagents. The silyl-protected guanosine **2** was synthesized from the guanosine derivative **1** in 2 steps (Scheme 2). After selectively protecting both the 5′ and 3′ hydroxy groups, a specific modification of the 2′ hydroxyl group with *tert*-butyldimethylsilyl group (TBDMS) was possible, yielding **2** (44,45). Subsequently, the NPOM caging group was installed through alkylkation of the N1 position of the guanine ring with **3** (46) in the presence of 1,8-diazabicyclo[5.4.0]undec-7-ene (DBU) in dimethylformamide (DMF), affording **4** in 79% yield. Selective deprotection of the 3′ and 5′ hydroxyl groups was achieved with HF-pyridine at 0°C, resulting in the caged nucleoside **5** in 89% yield. Installation of the DMT group at the 5′ position of **5** with dimethoxytrityl chloride (DMTCl) provided **6** in 83% yield, followed by the activation of the 3′ hydroxy group with 2-cyanoethyl-*N,N*-diisopropyl-chlorophosphoramidite in THF, delivering the NPOM-caged guanosine phosphoramidite **7** in 56% yield.

**Scheme 3.**
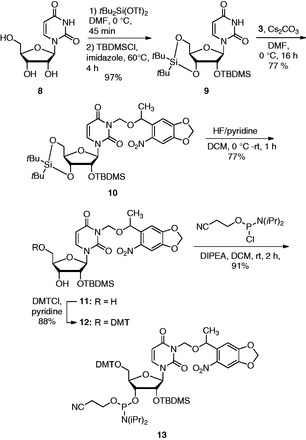
Synthesis of the NPOM-caged uridine phosphoramidite **13** from **8**. Dimethylformamide (DMF), *tert*-butyldimethylsilyl chloride (TBDMSCl), dichloromethane (DCM), di(*p*-methoxyphenyl)phenyl-methyl chloride (DMTCl), tetrahydrofuran (THF) and *N,N*-diisopropylethylamine (DIPEA).

To expand the flexibility of this RNA light-activation approach by increasing the number of potential caging sites, the NPOM-caged uridine phosphoramidite **11** was designed (Scheme 3). The silyl-protected uridine **9** was synthesized from uridine (**8**) in 97% yield. The nucleobase in **9** was then reacted with NPOM chloride (**3**) in the presence of Cs_2_CO_3_ in DMF, producing **10** in 77% yield. Selective removal of the 5′ and 3′ hydroxy protecting group furnished the caged uridine nucleoside **11** in 77% yield. The 5′ hydroxy group was then readily converted to the DMT ether **12** in 88% yield, and the 3′ hydroxy group was reacted with 2-cyanoethyl-*N,N*-diisopropylchlorophosphoramidite in *N,N*-diisopropylethylamine (DIPEA) and DCM in 91% yield to produce the NPOM-caged uridine phosphoramidite **13**.

### Polymerization and hybridization of NPOM-caged siRNA reagents

**Figure 1.**
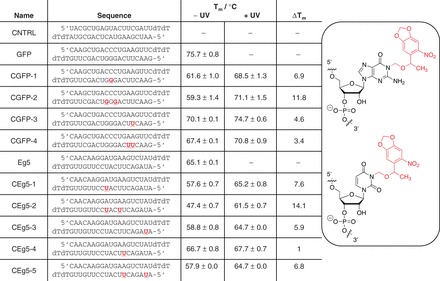
Sequences and T_m_ of caged siRNAs. Bold and underLined **G** denotes a caged guanosine nucleotide from the incorporation of **7** and a bold and underLined **U** denotes a caged uridine nucleotide from the incorporation of **13**. Standard deviations were calculated form three individual experiments.

After successful synthesis of both RNA phosphoramidites **7** and **13**, caged antisense strands were synthesized by incorporating the NPOM-caged nucleotides into synthetic siRNAs using standard RNA synthesis conditions on an Applied Biosystems DNA/RNA synthesizer. Based on Rana’s and Heckel’s chemical modification study of siRNAs (35,47), caged RNA phosphoramidites were incorporated into positions flanking the site of mRNA cleavage, nucleotides 9–11 (Figure 1). These previous studies (35,47) hypothesized that the caged nucleobase creates a bulge within the siRNA/mRNA duplex by site-specifically inhibiting Watson–Crick base pair formation without completely preventing hybridization of the two strands. This perturbation of the A-form conformation of the RNA interferes with the argonaute cleavage mechanism. In an alternate approach, caged uridine nucleotides were installed within the seed region, nucleotides 2–8, of the siRNA to prevent recognition of the target mRNA. In all cases, the caged siRNA sequences were designed to still maintain an RNA duplex with their complement at 37°C, as confirmed by T_m_ measurements (Figure 1 and Supplementary Information). The non-modified GFP siRNA duplex exhibits a T_m_ of 76°C. The addition of a single caged guanosine nucleotide at position 10 of the antisense strand CGFP-1 displayed a T_m_ of 62°C, 14°C lower than the non-caged GFP siRNA duplex. Therefore, while hybridization was observed, the RNA duplex was slightly perturbed resulting in a decreased T_m_. When two caged guanosine nucleotides were installed at positions 9 and 11 of the antisense strand CGFP-2, a similar effect was observed and the T_m_ decreased to 59°C. The incorporation of caged uridine nucleotides within the seed region did not affect the T_m_ to the same extent as the incorporation of caging groups closer to the center of the RNA antisense strand. For example, installation of the NPOM-caged uridine at nucleotide 5 in CGFP-3 led to a T_m_ of 70°C, only 6°C lower than the non-caged GFP siRNA. The incorporation of two caged uridine nucleotides at positions 5 and 6 in the antisense strand CGFP-4 provided a T_m_ of 67°C, indicating that the addition of a second caging group in the seed region only minimally induces further disruption of hybridization of the entire 19-mer. These results are comparable to previously caged double-stranded oligonucleotides in that the installation of one or two caged nucelobases can have a negligible effect on the T_m_ of an oligomer. Complete inhibition of duplex formation would require a caging group every five or six nucleobases (10,48). Here, all synthesized siRNA duplexes maintained a desired hybridization at 37°C despite the incorporation of 1–3 caged nucleotides. As expected, UV-induced caging group removal yielded double-stranded RNA molecules that displayed increased T_m_ comparable with the T_m_ of the non-caged GFP siRNA (Figure 1). The observed slightly lower T_m_ after UV exposure compared with the non-caged duplex could be the result of incomplete deprotection of the bulk sample.

In a design strategy following the GFP siRNA, NPOM-caged uridine phosphoramidites were incorporated at the cleavage site and the seed region of the antisense strand of the Eg5 siRNA and T_m_ were measured to ensure hybridization (Figure 1). The installation of a single caging group at nucleotide 11 of the antisense strand CEg5-1 resulted in a T_m_ of 58°C, displaying only a 7°C decrease in T_m_ compared with the Eg5 siRNA, which had a T_m_ of 65°C. The incorporation of two NPOM-caged uridine residues at nucleotides 8 and 11 in CEg5-2 resulted in a T_m_ of 48°C. Introduction of a single NPOM-caged uridine nucleotide within the seed regions of CEg5-3 and CEg5-4 resulted in a T_m_ of 59 and 64°C, respectively. The installation of two NPOM-caged uridine nucleotides within the seed region of the siRNA (CEg5-5) led to a T_m_ of 58°C. As observed for the GFP siRNA reagents, the presence of NPOM-caged uridine nucleotides in the Eg5 siRNA sequence resulted in only a slight perturbation of the RNA:RNA duplex structure without loss of hybridization at 37°C. After UV irradiation, the caging groups are removed, and increased T_m_, comparable to that of the non-caged Eg5 siRNA, were observed. No significant differences in T_m_ were observed for the nucleobase-caging of uridine versus guanosine, supporting the generality and predictability of our oligonucleotide light-activation approach.

### Light-activation of siRNA reagents targeting GFP

**Figure 2.**
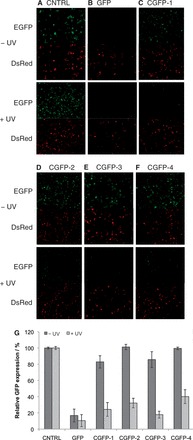
Photoactivated RNA interference in mammalian cells. HEK 293T cells were transfected with pEGFP-N1, pDsRed-N1 monomer and siRNA oligonucleotides. Cells were irradiated for 5 min (25 W, 365 nm) or kept in the dark. (**A–F**) Cells were imaged after 48 h. The GFP channel is shown above the DsRed channel. (**G**) After a 48-h incubation, the cells were trypsinized and analyzed by flow cytometry. The number of cells expressing both GFP and DsRed was normalized to the number of cells expressing only DsRed. Standard deviations were calculated from three individual experiments.

To test the activity of the caged siRNAs in mammalian cell culture, we first targeted the reporter gene GFP and used DsRed as a transfection control for fluorescent signal normalization. HEK 293T cells were co-transfected with pEGFP-N1, pDsRed-N1 monomer and the corresponding caged and non-caged duplex siRNA. After transfection, the cells were either irradiated for 5 min (365 nm, 25 W) or kept in the dark, followed by incubation for 48 h. The cells were then imaged and GFP and DsRed expression was quantified using flow cytometry. The number of cells expressing both GFP and DsRed was normalized to cells expressing only DsRed. A scrambled siRNA was used as a negative control (CNTRL) and, as expected, showed no inhibition of GFP expression (Figure 2A and G). Cells treated with the GFP siRNA (positive control) displayed a 90% reduction of GFP expression (Figure 2B). In the presence of the single NPOM-caged guanosine nucleotide in CGFP-1, 82% GFP expression was observed (Figure 2C), indicating that the caged siRNA reagent was inactive due to the presence of the caging group close to the argonaute cleavage site. The incorporation of two caged guanosine nucleotides in CGFP-2 resulted in complete inhibition of siRNA activity before irradiation, as GFP expression levels identical with the negative control were observed (Figure 2D). After a brief irradiation with UV light (365 nm), the caging groups were removed, activating CGFP-1 and CGFP-2, and resulting in the silencing of GFP expression to ∼20–30% of native levels (comparable to the non-caged analogs). An irradiation time course was performed and no difference in GFP silencing was observed between a 2-min and a 10-min irradiation (see Supporting Information). Thus, by installing the NPOM-caged guanosine nucleotides within the central region of the siRNA duplex, mRNA silencing by argonaute is inhibited until light-induced removal of the caging group(s), effectively creating UV-activated siRNA reagents.

The seed region of non-coding RNAs, like siRNAs and miRNAs, is essential in the recognition of their target genes (49). Therefore, NPOM-caged uridine nucleotides were introduced within the seed region of siRNAs to determine whether this approach is also viable for the development of light-activatable siRNAs by specifically disrupting the siRNA:mRNA interaction and not necessarily the mRNA-cleavage activity of RISC. Having two validated approaches to the light-regulation of RNA interference will provide greater flexibility in the design and synthesis of caged non-coding RNA reagents and will allow for the generation of light-activated RNA molecules that do not rely on argonaute cleavage, e.g. miRNAs. Gratifyingly, the caged siRNA duplex CGFP-3, bearing one caged uridine in the seed region, was virtually inactive in HEK 293T cells, as 82% of GFP expression was observed (Figure 2E). On addition of a second caging group in CGFP-4, complete inactivation of siRNA activity was observed, identical to the negative control siRNA (Figure 2F). After UV exposure, the silencing activity of the siRNAs was restored with only 19 and 36% GFP expression observed for CGFP-3 and CGFP-4, respectively (Figure 2G). These results indicate that two different caging approaches—installation of caging groups close to the argonaute cleavage site or in the RNA seed region—can be used to generated light-activated siRNA reagents with excellent ‘off’ to ‘on’ switching ratios, resembling those of negative and positive control reagents.

### Light-activation of siRNA reagents targeting Eg5

**Figure 3.**
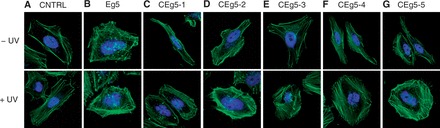
Optochemical activation of Eg5 siRNA in HeLa cells. HeLa cells were transfected with caged and non-caged siRNAs (40 pmol). The cells were irradiated (5 min, 25 W, 365 nm) and incubated at 37°C, 5% CO_2_ for 48 h. (**A–G**) The cells were fixed and stained with Alexa Fluor 488 phalloidin (green) and DAPI (blue). The cells were imaged on a Zeiss LSM 710 confocal microscope using a 40× oil objective and Alexa Fluor 488 and DAPI-specific lasers (488 nm multiline argon and 405 nm diode).

Next, the light-activation of caged siRNA reagents targeting the endogenous Eg5 gene was tested in mammalian cell culture. HeLa cells were transfected with the caged Eg5 siRNAs (50), and the cells were either briefly irradiated with UV light (5 min, 25 W, 365 nm) or kept in the dark. After a 48-h incubation, the cells were fixed, stained with Alexa Fluor 488 phalloidin (to label actin filaments) and DAPI (to label nuclei) and imaged by confocal microscopy. The negative control siRNA sequence displayed no obvious change in phenotype (Figure 3A) in the presence or absence of UV irradiation. As expected, Eg5 siRNA transfected cells led to a binucleated phenotype due to Eg5 silencing and cell cycle arrest within mitosis (50,51), with most cells showing 2–3 nuclei (Figure 3B). CEg5-1 and CEg5-2 containing NPOM-caged uridine nucleotides within the central argonaute-cleavage region of the siRNA displayed a phenotype identical to the negative control when kept in the dark, indicating that the caged Eg5 siRNA reagents are functionally inactive (Figure 3C and D). After a brief irradiation (5 min, 25 W, 365 nm), binucleated cells were observed, showing a phenotype identical to cells treated with the Eg5 siRNA positive control. This indicates that the siRNA reagents were optochemically activated for suppression of Eg5 expression. Moreover, the incorporation of a single caged NPOM-caged uridine nucleotide within the seed region, as in CEg5-3 and CEg5-4, functionally inactivated the siRNA, leading exclusively to a normal cell phenotype (Figure 3E and F, respectively). Upon irradiation, the Eg5-knockdown phenotype is observed as a result of light-activation of the siRNA molecules. As expected, the introduction of a second NPOM-caged uridine nucleotide within the seed region of CEg5-5 also fully inactivated the siRNA (Figure 3G), and activity was again restored through UV exposure. These results demonstrate, for the first time, the application of caged siRNA in the light-induced silencing of an endogenous gene.

**Figure 4.**
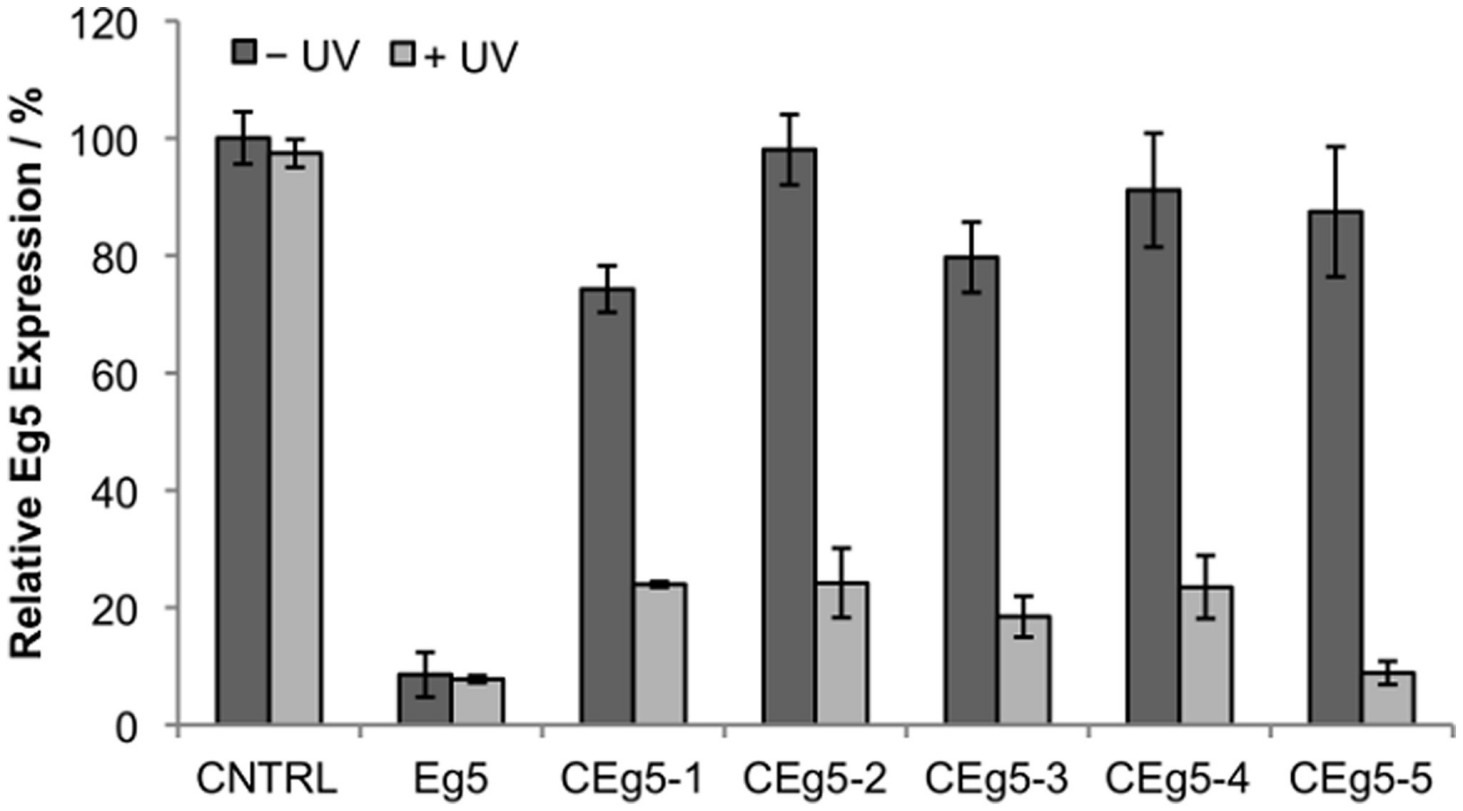
Quantification of photoactivation of Eg5 siRNA. HeLa cells were transfected with siRNAs, and after 48-h incubation, the RNA was extracted and quantitative real-time PCR analysis was performed. Eg5 expression was normalized to the expression of the GAPDH housekeeping gene, and the negative control was set to 100% Eg5 expression. Error bars represent standard deviations from three independent experiments.

To quantify the optochemically induced Eg5 gene silencing, total RNA isolated from cells transfected with the corresponding siRNAs and either irradiated or kept in the dark was subjected to quantitative real-time PCR analysis (37). Eg5 expression was normalized to the endogenous housekeeping gene GAPDH and the inactive negative control siRNA was set to 100%. In agreement with the previous phenotypic observations, the negative control siRNA (CNTRL) did not inhibit Eg5 expression, whereas the Eg5 positive control siRNA silenced expression to 8% (Figure 4). The incorporation of an NPOM-caged uridine nucleotide within the central argonaute-cleavage region of the siRNA (CEg5-1) renders the siRNA mostly inactive with 74% Eg5 expression detected. The addition of a second caged nucleotide within this region (CEg5-2) completely inactivates the siRNA, with 98% Eg5 expression observed. UV irradiation restores Eg5 siRNA activity of both CEg5-1 and CEg5-2 to the same extent, with only 24% Eg5 mRNA levels detected. The introduction of NPOM-caged uridine nucleotides within the seed region of the siRNA, as in CEg5-3 and Eg5-4, also led to the inactivation of the siRNA with 79 and 91% of Eg5 expression observed, respectively. After a brief UV irradiation, the caging groups are cleaved, activating the siRNA, and leading to the inhibition of Eg5 with only 18 and 24% of residual Eg5 expression observed for CEg5-3 and CEg5-4, respectively. CEg5-5 follows a similar trend with 87% Eg5 expression before UV irradiation, indicating a virtually inactive siRNA agent, and restoration of Eg5 silencing to native levels through irradiation, with only 8% Eg5 expression detected after UV exposure.

## DISCUSSION

The development of light-regulated siRNA molecules affords precise activation of RNA interference with widespread applications in the regulation and investigation of gene function and expression. Here, we set out to achieve full inactivation of siRNA function using a robust nucleobase caging technology and demonstrate that this methodology can be applied to the optochemical regulation of both exogenous and endogenous genes in mammalian cells.

To achieve this goal, synthetic routes to two new NPOM-caged phosphoramidites, the NPOM-caged uridine **7** and the NPOM-caged guanosine **13**, were developed. Guided by the established RNAi mechanism (52), siRNA sequence analysis (47) and RISC structural studies (30), the two phosphoramidites were site-specifically incorporated at defined residues within siRNA oligonucleotides. Two different strategies were tested: (i) By installing caged uridine nucleotides within the seed region of the siRNA molecule (nucleotides 2–8), it was expected that the caging groups would suppress the recognition of the target mRNA while maintaining stability of the siRNA duplex under physiological conditions. (ii) Caged guanosine and uridine nucleotides were introduced within the central argonaute-cleavage region of different siRNA reagents. Several studies have shown that base pair mismatches and chemical modification at nucleotide positions 10–11 of an siRNA antisense strand diminishes RNAi activity (32,47,53).

T_m_ of the caged RNA oligonucleotides hybridized to their complementary sequences were measured to determine that the placement of the caging groups will inhibit only essential RNA:mRNA hydrogen bonding interactions or protein function within RISC, but does not prevent hybridization of the siRNA duplexes. Gratifyingly, the introduction of only 1–2 caging groups does not prevent RNA:RNA hybridization at physiological temperatures and only slightly lowers the T_m_ of the 19 nucleotide siRNA duplexes by 1–14°C. A greater decrease in T_m_ is observed when the caging group is placed within the central region of the duplex, in agreement with previous studies on caged DNA:DNA duplexes (48).

The caged siRNA molecules were subsequently tested in mammalian cell culture for the photoregulation of GFP reporter expression. The installation of 1–2 NPOM-caging groups within the central region (positions 9–11) of the siRNAs CGFP-1 and CGFP-2 completely inhibited siRNA activity (2–17%), presumably by preventing the cleavage mechanism within the RISC complex. Furthermore, brief UV irradiation restored siRNA function to 75–80% when compared to the negative control siRNA reagent (see Figure 2). Caging the central region of the siRNA is an excellent strategy to control siRNA function with light; however, not all siRNAs induce RNA cleavage (41) and caging the seed region may provide a more universal approach to the optochemical regulation of non-coding RNA function. The seed region plays a very important role in the recognition of the mRNA target. Thus, in a second light-regulation approach, NPOM-caged nucleotides were synthetically introduced within positions 2–7 of the seed region of the siRNA sequences (see Figure 1). The presence of 1–2 caging groups inhibited RNAi activity until UV irradiation removed the caging groups and restored active siRNA molecules. For example, CGFP-3 siRNA shows excellent photoswitching ability from an inactive siRNA molecule to an active siRNA reagent through UV exposure, inducing gene silencing comparable to a non-caged GFP siRNA. The caged nucleotides were also incorporated into an siRNA reagent targeting an endogenous gene, Eg5. The photoactivation of Eg5 inhibition confirmed the findings from the GFP reporter studies and demonstrates for the first time that these photochemical tools can be used for the regulation of endogenous genes.

Interestingly, Mikat *et al.* previously reported an NPP-caged deoxyguanosine nucleotide that was incorporated at the third position of the antisense strand (within the seed region) of GFP siRNA that still displayed substantial RNA-silencing activity (35). This is in contrast to the findings presented here where the incorporation of an NPOM-caged uridine at the fourth and fifth positions of the antisense strand (CGFP-3 and CGFP-4) resulted in 80–90% inhibition of siRNA activity. In addition, the caging groups on the siRNA duplexes maintain stability for at least 48 h, in contrast to previously reported, less stable NPP-caged siRNAs that regained silencing activity after a 28-h incubation in mammalian cells in the dark (35). The difference in the caged siRNA activity may be the result of various effects, including the use of different caging group structures and their different installation on the nucleobases (N-NPOM versus O-NPP). The higher stability of the NPOM group installed at the uridine N^3^ and the guanosine N^1^ was also observed during oligonucleotide synthesis. The fact that actual caged RNA nucleotides are used here, rather than caged DNA nucleotides, may also have an effect.

Other previously reported caged siRNA reagents were generated by installing DMNPE groups on the terminal phosphates of dsRNA to block dicer activity (54). Additionally, the DMNPE caging group has been statistically incorporated into siRNA (55) and 2′-fluoro-substituted siRNA reagents (34). In all of these studies, complete inhibition of siRNA activity was not achieved, as 15–40% of residual siRNA activity was observed. However, full siRNA function (80–100%) was restored after UV irradiation. One limitation to these light-activated RNAi reagents is the statistical incorporation of the caging group, which may account for the ‘leakiness’ of the caged siRNAs. Recently, Jain *et al.* introduced more sterically demanding cyclododecyl DMNPE caging groups to the four termini of the double-stranded siRNA and achieved complete inhibition of silencing activity until UV exposure.(33) Although this methodology provides a means to regulate RNAi function with light, no sequence/structure-function information is gained from the placement of the caging groups. Through the site-specific incorporation of caged nucleobases, one to two essential nucleotides can be used to photoregulate siRNA activity with no background leakiness and full restoration of siRNA function after UV irradiation (see Figures 2G and 4). Moreover, depending on the position of the caged nucleotide in the RNA sequence, different functions—RISC activity versus seed recognition—may be optochemically regulated to control gene silencing by siRNAs and miRNAs.

In summary, we have developed novel light-activated siRNA reagents through the site-specific introduction of new nucleobase-caged RNA nucleotides into oligonucleotides. Two new NPOM-caged RNA phosphoramidites, bearing caging groups with red-shifted absorption maxima, were synthesized and were successfully incorporated into RNA using standard automated solid-phase oligonucleotide synthesis. Hybridization studies showed that the presence of one or two caging groups led to a slightly lower T_m_ of the siRNA duplex but did not prevent RNA:RNA hybridization. The activity and light-regulation of the caged siRNAs were assayed with a reporter gene, GFP, and an endogenous gene, Eg5. Through the incorporation of one or two caging groups within the central nucleotides 8–11 of the antisense strand of the siRNA, the siRNA reagent was rendered completely inactive, presumably due to the temporary blocking of argonaute function (56). However, after irradiation, the caging groups were removed, and the siRNAs were fully functional, leading to efficient gene silencing in mammalian cells. A second successful caging strategy that involves incorporation of caged RNA nucleotides into the seed region of siRNAs should be easily adapted to the optochemical control of miRNAs, as those also rely on the seed region for recognition of the target mRNAs but do not induce argonaute-catalyzed mRNA cleavage (30). These nucleobase-caged RNA molecules can be used to study the spatial and temporal dynamics of miRNA function. The dynamic behavior of miRNAs is becoming increasingly evident in developmental processes, especially in the brain (57), and the developed approach will provide an excellent tool for localized cellular and sub-cellular activation of miRNA function. Additionally, the kinetics of the siRNA and miRNA pathways can be evaluated, as the time required for RNA transfection can be separated from the time needed for a functional output. The light-activation strategy presented here is not only expected to allow for silencing of a wide range of genes in mammalian tissue culture, but also in multicellular organisms with a functional siRNA or miRNA pathway, such as plants (58), *Caenorhabditis elegans* (59), drosophila (60) and mice (61).

## SUPPLEMENTARY DATA

Supplementary Data are available at NAR Online.

## FUNDING

National Institutes of Health [R01GM079114]; the Beckman Foundation (Beckman Young Investigator Award to A.D.); Research Corporation (Cottrell Scholar Award to A.D.). Funding for open access: NCSU.

*Conflict of interest statement*. None declared.

## Supplementary Material

Supplementary Data
