# Supplementary material for: Optochemical control of RNA interference in mammalian cells
Source: Nucleic Acids Res. 2013 Sep 10;41(22):10518–28. doi: 10.1093/nar/gkt806 (PMC3905849; doi:10.1093/nar/gkt806)
Supplement: Supplementary Data [file supp_41_22_10518__index.html]

Optochemical control of RNA interference in mammalian cells — Optochemical control of RNA interference in mammalian cells — Supplementary Data 

# Optochemical control of RNA interference in mammalian cells

## Supplementary Data

files

**Files in this Data Supplement:**

- Supplementary Data - pdf file
