# Supplementary material for: Optochemical control of RNA interference in mammalian cells
Source: Nucleic Acids Res. 2013 Sep 10;41(22):10518–28. doi: 10.1093/nar/gkt806 (PMC3905849; doi:10.1093/nar/gkt806)
Supplement: Supplementary Data [file supp_gkt806_nar-01360-y-2013-File001.pdf]

Supporting Information

Optochemical Control of RNA Interference in Mammalian Cells

Jeane M. Govan[1], Douglas D. Young[2], Hrvoje Lusic[1], Qingyang Liu[1], Mark O. Lively[3], and Alexander Deiters[1,4,*]

[1] Department of Chemistry, North Carolina State University, Raleigh, NC 27695

[2] Department of Chemistry, College of William & Mary, Williamsburg, VA 32187

[3] Center for Structural Biology, Wake Forest University School of Medicine, Winston-Salem, NC 27157

[4] Department of Chemistry, University of Pittsburgh, Pittsburgh, PA 15260

To whom correspondence should be addressed: Tel: (+1) 412-624-8200; Fax: (+1) 412-624-8611; Email: deiters@pitt.edu

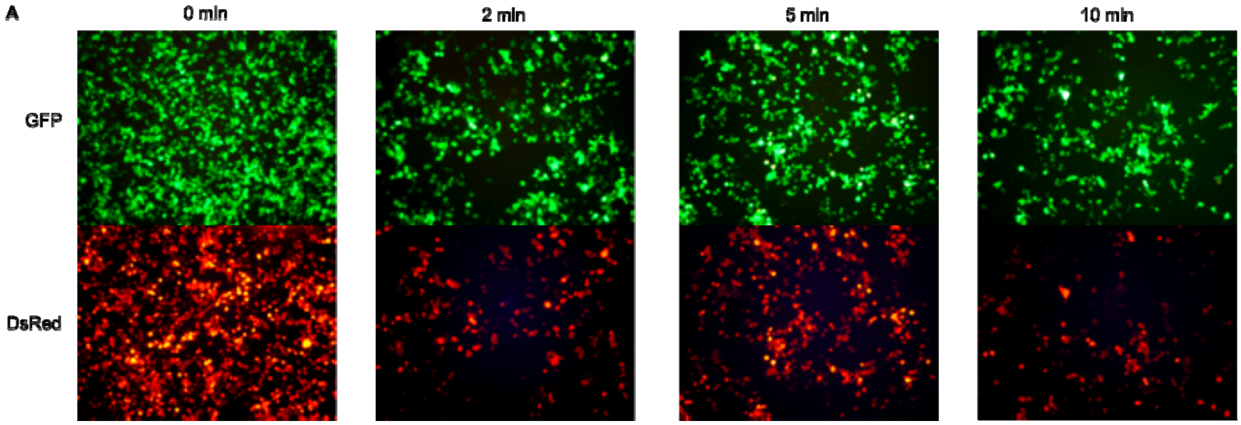

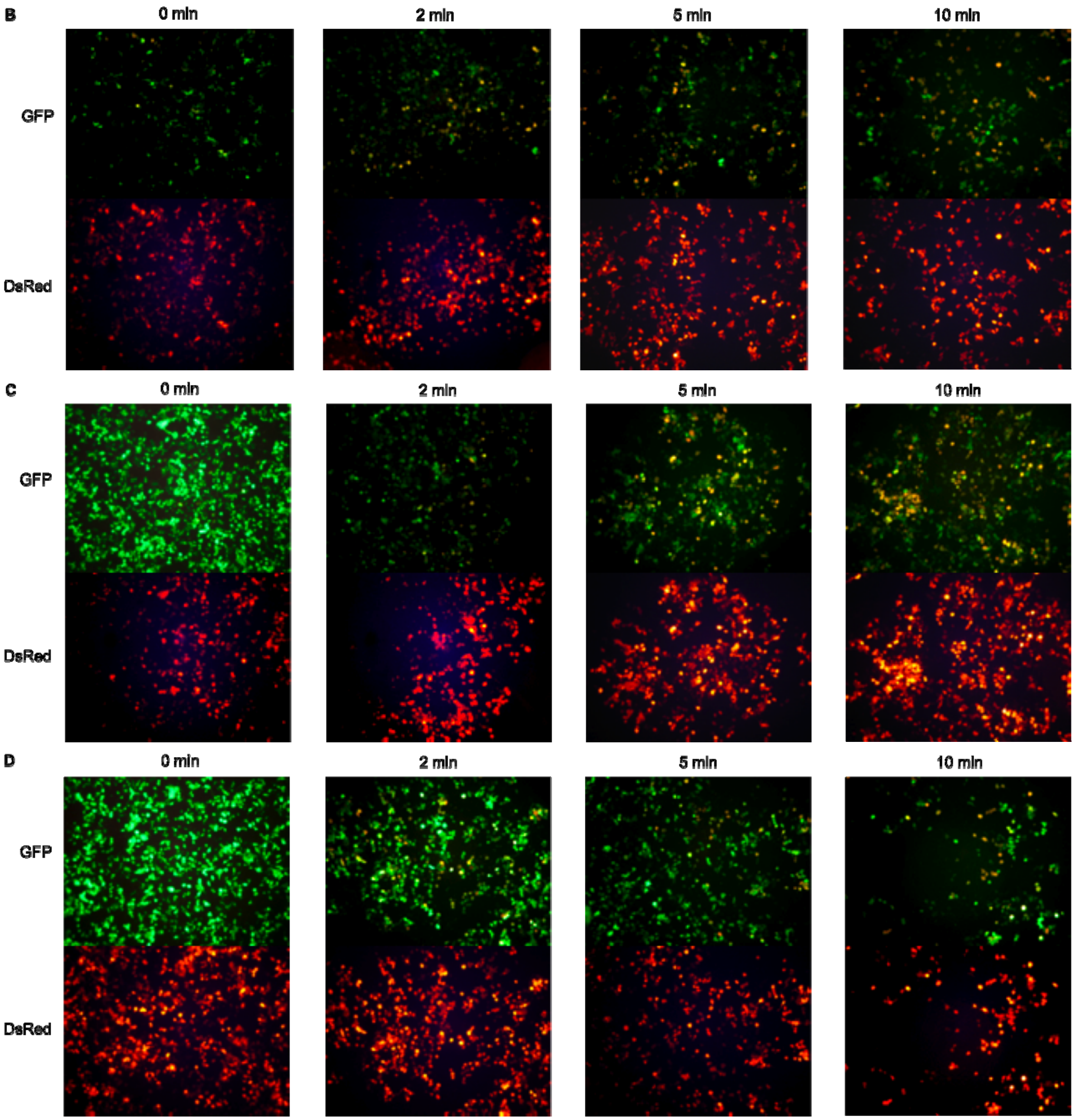

Supporting Information Figure 1. Irradiation time course of HEK 293T cells transfected with caged GFP siRNA. HEK 293T cells were transfected with pEGFP-N1, pDsRed-N1 monomer, and siRNA oligonucleotides. Cells were irradiated for 0, 2, 5, or 10 min (25 W, 365 nm). Cells were imaged after 48 h on a Jenco inverted microscope. The GFP channel is shown above the DsRed channel. **A.** CNTRL siRNA **B.** GFP **C.** CGFP-1 **D.** CGFP-2

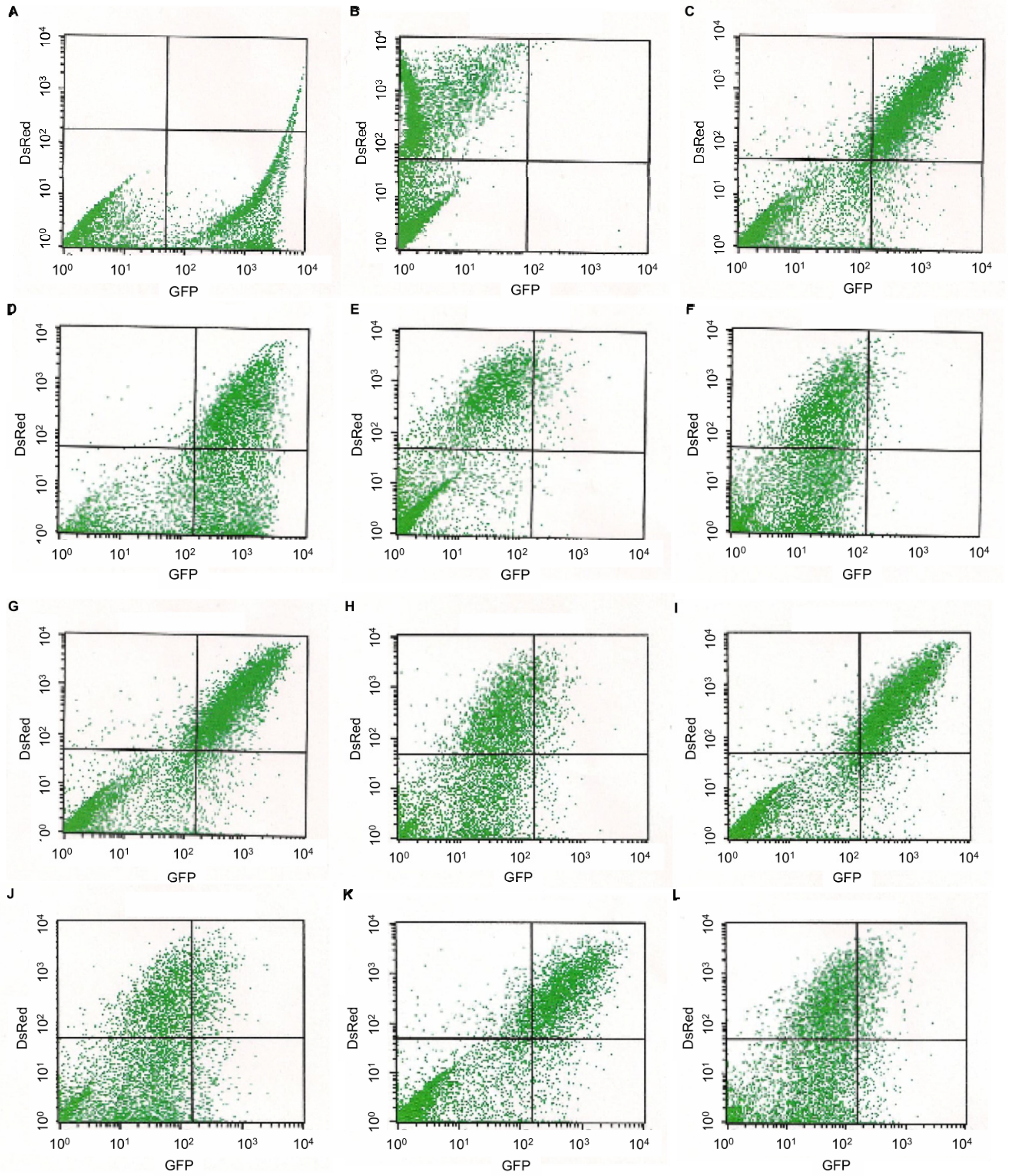
A
B
C
D
E
F
G
H
I
J
K
L
DsRed
GFP
10⁰
10¹
10²
10³
10⁴

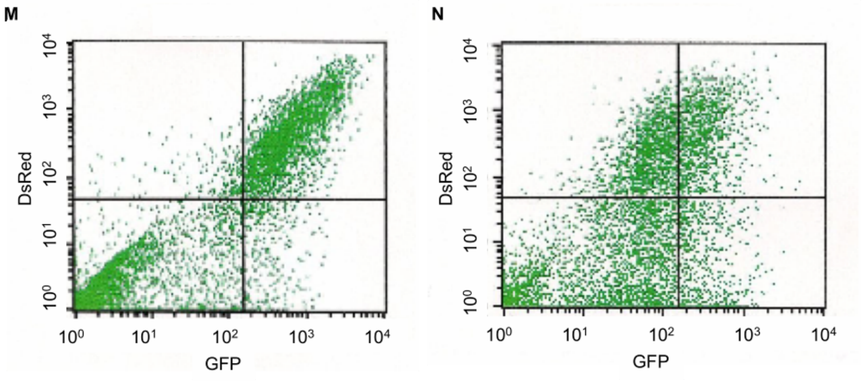

Supporting Information Figure 2. FACS plots of HEK 293T cells transfected with caged GFP siRNA. HEK 293T cells were transfected with pEGFP-N1, pDsRed-N1 monomer, and siRNA oligonucleotides. Cells were irradiated for 5 min (25 W, 365 nm) or kept in the dark. After a 48 h incubation, the cells were trypsinized and analyzed by FACS. Represnetative plots are shown. **A.** HEK 293T cells transfected with pEGFP-N1 **B.** HEK 293T cells transfected with pDsRed-N1 monomer **C.** CNTRL siRNA − UV **D.** CNTRL siRNA + UV **E.** GFP-1 − UV **F.** GFP-1 + UV **G.** CGFP-1− UV **H.** CGFP-1 + UV **I.** CGFP-2 − UV **J.** CGFP-2 + UV **K.** CGFP-3 − UV **L.** CGFP-3 + UV **M.** CGFP-4 − UV **N.** CGFP-4 + UV.

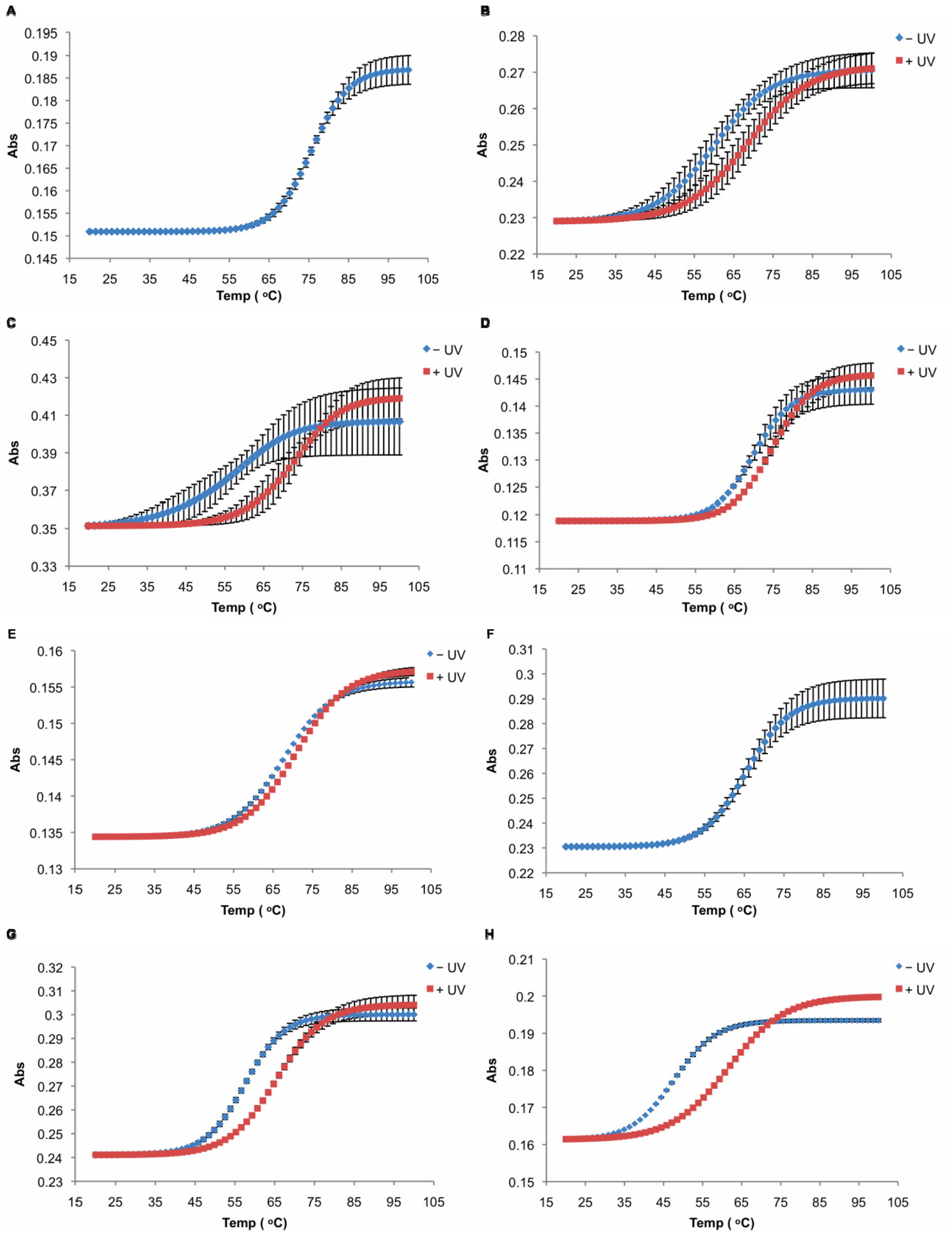
A
B
C
D
E
F
G
H
Abs
Temp (ºC)
– UV
+ UV

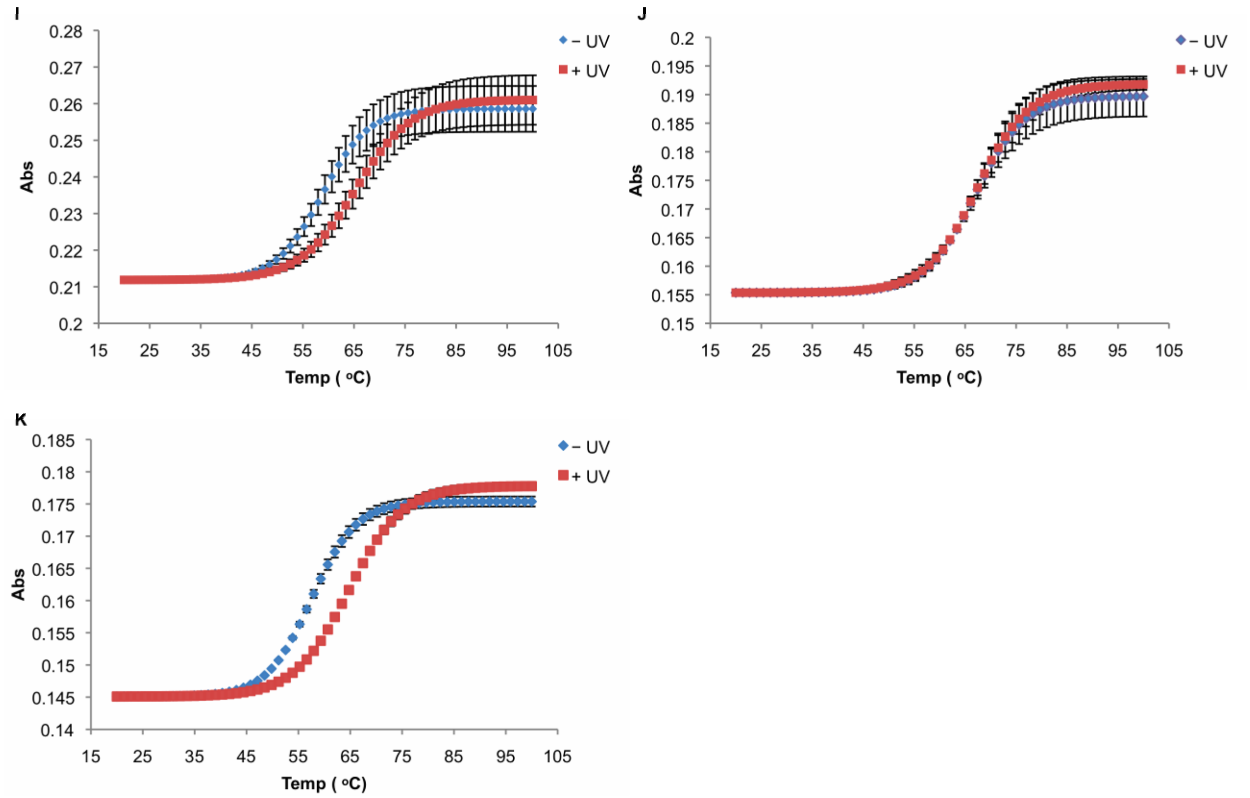

Supporting Information Figure 3. Melt curves of caged siRNAs. The two complementary strands of siRNAs (1 μM) were incubated in 0.15 M NaCl, 0.05 M NaH_2PO_4, pH 7.2 buffer. The samples were protected from light or irradiated at 365 nm with a UV transilluminator for 20 min, heated to 100 °C for 2 min, and then cooled to 20 °C at a rate of 2 °C/min, held at 20 °C for 5 min, then heated to 100 °C at a rate of 2 °C/min. Absorbance was recorded at 260 nm every 1 °C. The T_m was determined by the maximum of the first derivative of the absorbance vs. temperature plot. Standard deviations were calculated form three individual experiments. **A.** GFP **B.** CGFP-1 **C.** CGFP-2 **D.** CGFP-3 **E.** CGFP-4 **F.** Eg5 **G.** CEg5-1 **H.** CEg5-2 **I.** CEg5-3 **J.** CEg5-4 **K.** CEg5-5

Synthetic Protocols

(*E*)-*N*'-(9-((4a*R*,6*R*,7*R*,7a*R*)-2,2-di-*tert*-butyl-7-((*tert*-butyldimethylsilyl)oxy)tetrahydro-4*H*-furo[3,2-*d*][1,3,2]dioxasilin-6-yl)-1-((1-(6-nitrobenzo[*d*][1,3]dioxol-5-yl)ethoxy)methyl)-6-oxo-6,9-dihydro-1*H*-purin-2-yl)-*N,N*-dimethylformimidamide (4). The guanosine derivative **2** was synthesized as previously reported.(44) The NPOM caging group **3** was prepared according to the previously described synthesis.(45) The silyl protected guanosine **2** (100 mg, 0.17 mmol) was dissolved in DMF (1 mL) with DBU (38 μL, 0.25 mmol). NPOM-Cl **3** (64 mg, 0.25 mmol) dissolved in DMF (0.5 mL) was added dropwise, and the solution was stirred for 12 h at room temperature. EtOAc (10 mL) was added and the organic layer was washed with saturated aqueous $NaHCO_3$, water, and brine (10 mL each). The organic layer was dried over anhydrous Na_2SO_4, filtered, and the solvent was evaporated. Purification of the crude product by silica gel chromatography using a hexanes/EtOAc gradient (70:30 to 50:50) containing 2% TEA afforded **4** as a yellow foam in 79% yield (108 mg, 0.13 mmol). ^{1}H NMR (300 MHz, $CDCl_3$): δ = 0.11 (s, 6 H), 0.90 (s, 9 H), 1.01-1.07 (m, 18 H), 1.46 (d, *J* = 6.3 Hz, 3 H), 3.04 (s, 3 H), 3.17 (s, 3 H), 3.95-4.09 (m, 3 H), 4.42-4.49 (m, 2 H), 5.37-5.42 (m, 1 H), 5.63-5.98 (m, 5 H), 7.18-7.31 (m, 2 H), 7.51 (s, 1 H), 8.32 (s, 1 H). ^{13}C NMR (75 MHz, $CDCl_3$): δ = –4.7, –4.1. 18.6, 20.6, 23.0, 24.1, 26.1, 27.2, 27.7, 35.4, 41.5, 68.1, 74.5, 75.5, 76.2, 76.6, 91.0, 91.6, 103.0, 104.5, 107.2, 120.4, 135.4, 136.1, 139.3, 141.2, 146.8, 147.6, 152.5, 157.1, 157.8. HRMS-LC: *m/z* calcd for $C_{37}H_{57}N_7O_{10}Si_2$ $[M+H]^+$: 816.3783; found: 816.3774.

(*E*)-*N*'-(9-((2*R*,3*R*,4*R*,5*R*)-3-((*tert*-butyldimethylsilyl)oxy)-4-hydroxy-5-(hydroxymethyl)tetrahydrofuran-2-yl)-1-((1-(6-nitrobenzo[*d*][1,3]dioxol-5-yl)ethoxy)methyl)-6-oxo-6,9-dihydro-1*H*-purin-2-yl)-*N*,*N*-dimethylformimidamide (5). The NPOM-guanosine **4** (100 mg, 0.12 mmol) was dissolved in DCM (0.5 mL) and the solution was cooled to 0 ºC in a plastic vessel. A solution of HF-pyridine (13 μL, 70% HF-pyridine, 0.5 mmol) diluted with pyridine (80 μL) was slowly added, and the reaction mixture was stirred for 2 h at 0 ºC. The reaction mixture was subsequently quenched with saturated aqueous $NaHCO_3$ (5 mL) and diluted with DCM (5 mL). The organic layer was washed with water (5 mL), dried over anhydrous Na_2SO_4, filtered and concentrated in vacuum. Purification of the crude product by silica gel chromatography using $CHCl_3$/MeOH (98:2) containing 2% TEA, afforded **5** as a yellow foam in 89% yield (72 mg, 0.11 mmol). ^{1}H NMR (300 MHz, $CDCl_3$): δ = –0.28 (s, 1.5 H), –0.24 (s, 1.5 H), –0.16 (s, 1.5 H), 0.12 (s, 1.5 H), 0.77 (s, 6 H), 0.90 (s, 3 H), 1.39 (d, *J* = 5.7 Hz, 3 H), 2.95 (d, *J* = 6.3 Hz, 3 H), 3.13 (d, *J* = 6.0 Hz, 3 H), 3.62-4.10 (m, 3 H), 4.18-4.77 (m, 4 H), 5.26-5.37 (m, 1 H), 5.58-5.88 (m, 5 H), 6.94-7.04 (m, 1 H), 7.22-7.25 (m, 1 H), 7.65-7.70 (m, 0.5 H), 7.92-8.02 (m, 0.5 H), 8.13 (s, 0.5 H), 8.20 (s, 0.5 H). ^{13}C NMR (75 MHz, $CDCl_3$): δ = –4.9, –4.4, 18.1, 24.0, 25.9, 35.2, 41.5, 62.3, 71.6, 74.3, 75.1, 76.2, 85.9, 90.0, 103.1, 104.6, 106.8, 120.7, 138.1, 138.9, 139.0, 141.1, 146.9, 147.7, 152.3, 157.6, 157.9. HRMS-LC: *m/z* calcd for $C_{29}H_{41}N_7O_{10}Si$ $[M+H]^+$: 676.2762; found: 676.2758.

(*E*)-*N*'-(9-((2*R*,3*R*,4*R*,5*R*)-5-((bis(4-methoxyphenyl)(phenyl)methoxy)methyl)-3-((*tert*-butyldimethylsilyl)oxy)-4-hydroxytetrahydrofuran-2-yl)-1-((1-(6-nitrobenzo[*d*][1,3]dioxol-5-

yl)ethoxy)methyl)-6-oxo-6,9-dihydro-1*H*-purin-2-yl)-*N*,*N*-dimethylformimidamide (6). Dimethoxytrityl chloride (60 mg, 0.17 mmol) was added to a solution of NPOM-guanosine derivative **5** (100 mg, 0.15 mmol) and DMAP (spatula tip) in pyridine (1 mL) and the reaction mixture was stirred for 12 h at room temperature. The reaction mixture was quenched with MeOH (1 mL) and the solvents were evaporated. Purification of the residue by silica gel chromatography, using a $CHCl_3$/hexanes gradient (70:30-80:20) with 2% TEA, afforded **6** as a yellow foam in 83% yield (120 mg, 0.12 mmol). ^{1}H NMR (300 MHz, $CDCl_3$): δ = –0.19-(–0.12) (m, 1.5 H), –0.02-0.08 (m, 4.5 H), 0.81-0.89 (m, 9 H), 1.46 (t, *J* = 6.3 Hz, 3 H), 3.02-3.04 (m, 3 H), 3.09-3.14 (m, 3 H), 3.22-3.44 (m, 2 H), 3.76-3.79 (m, 6 H), 4.11-4.39 (m, 3 H), 4.62 (q, *J* = 5.4 Hz, 1 H), 5.35-5.46 (m, 1 H), 5.65-5.98 (m, 5 H), 6.80-6.85 (m, 4 H), 7.20-7.44 (m, 11 H), 7.73-7.78 (m, 1 H), 8.30 (s, 0.5 H), 8.38-8.41 (m, 0.5 H). ^{13}C NMR (75 MHz, $CDCl_3$): δ = –4.8, –4.4, 18.1, 24.1, 25.8, 35.4, 41.4, 55.5, 63.1, 63.9, 71.9, 75.3, 76.5, 84.0, 86.9, 87.9, 103.1, 104.4, 104.8, 107.1, 113.5, 120.1, 127.3, 128.2, 130.3, 135.7, 139.5, 141.1, 144.7, 146.8, 148.5, 152.6, 157.3, 157.8, 157.9, 158.9. HRMS-LC: *m/z* calcd for $C_{50}H_{59}N_7O_{12}Si$ $[M+H]^+$: 978.4069; found: 978.4064.

(2*R*,3*S*,4*S*,5*R*)-2-{[Bis(4-methoxyphenyl)(phenyl)methoxy]methyl}-4-(*tert*-butyldimethylsilyloxy)-5-{2-[(*E*)-(dimethylamino)methyleneamino]-1-{[1-(6-nitrobenzo[d][1,3]dioxol-5-yl)ethoxy]methyl}-6-oxo-1,6-dihydropurin-9-yl}-tetrahydrofuran-3-yl 2cyanoethyl diisopropylphosphoramidite (7). 2-cyanoethyl-*N,N*-diisopropyl-chlorophosphoramidite (45 μL, 0.2 mmol) was added to an ice-cold solution of **6** (100 mg, 0.1 mmol), 2,4,6-collidine (54 μL, 0.4 mmol), and *N*-methylimidazole (8 μL, 0.1 mmol) in THF (1 mL). The reaction mixture was stirred at room temperature for 2 h and then diluted with EtOAc (10 mL). The resulting mixture was washed with saturated aqueous $NaHCO_3$, water, and brine (10 mL each). The organic layer was dried over anhydrous Na_2SO_4 and concentrated to give an oily residue. Purification of the residue by sílica gel chromatography using hexanes/EtOAc (30:70) with 2% TEA afforded **7** as a yellow foam in 56% yield (62 mg, 0.056 mmol). ^{1}H NMR (300 MHz, $CDCl_3$): δ = –0.19-0.04 (m, 6 H), 0.77-1.15 (m, 21 H), 1.41-1.43 (m, 3 H), 2.07-2.62 (m, 2 H), 2.98-3.11 (m, 6 H), 3.20-3.67 (m, 5 H), 3.75 (s, 6 H), 4.11-4.63 (m, 4 H), 5.35-5.41(m, 1 H), 5.62-6.10 (m, 5 H), 3.68 (d, *J* = 6.0 Hz, 4 H), 7.10-7.50 (m,11 H), 7.75-7.84 (m, 1 H), 8.25-8.47 (m, 1 H). ^{13}C NMR (75 MHz, $CDCl_3$): δ = –4.8, –4.1, 18.1, 20.0, 24.1, 24.8, 25.9, 35.4, 41.4, 43.5, 55.5, 57.9, 63.5, 63.8, 72.2, 73.1, 76.5, 83.7, 84.7, 86.8, 103.1, 104.6, 104.8, 107.1, 113.5, 117.6, 120.1, 127.3, 128.2, 130.3, 135.7, 139.5, 141.1, 144.7, 146.9, 148.5, 152.6, 157.3, 157.8, 158.0, 158.8. ^{31}P NMR (121 MHz, $CDCl_3$): δ = 150.3, 150.5, 150.7, 151.3, 151.6, 151.7, 151.8.

1-((4a*R*,6*R*,7*R*,7a*R*)-2-(*tert*-butyl)-2-butyl-7-((*tert*-butyldimethylsilyl)oxy)tetrahydro-4*H*-furo[3,2-*d*][1,3,2]dioxasilin-6-yl)pyrimidine-2,4(1*H*,3*H*)-dione (9). Uridine **8** (2.0 g, 8.2 mmol) was co-evaporated with dry pyridine (2 x 10 mL), then dissolved in dry DMF (20 mL), and cooled to 0 ºC. (t-Bu)$_2$(OTf)$_2$Si (2.85 mL, 8.8 mmol), was added via a syringe pump over 45 min and the reaction mixture was stirred for another 45 min at 0 ºC. Imidazole (2.72 g, 40 mmol) was added, and the reaction was brought to room temperature, followed by the slow addition of *tert*-butyldimethylchlorosilane (1.578 g, 10.4 mmol). The reaction mixture was then heated to 60 ºC for 4 h, cooled to room temperature, and the

solvent was removed by rotatory evaporation. Water (60 mL) and chloroform (120 mL) were added, the layers were separated and the organic layer was washed with brine (2 x 30 mL) and dried with anhydrous Na_2SO_4, gravity filtered, and evaporated by rotatory evaporation. The oily residue was purified by column chromatography using $CHCl_3$/MeOH (100:1), delivering the product **9** as a white solid (3.9 g, 97%). ^{1}H NMR (400 MHz, $CDCl_3$): δ 0.14 (s, 3 H), 0.19 (s, 3 H), 0.93 (s, 9 H), 1.02-1.10 (m, 18 H), 3.85-3.88 (dd, J_a = 9.8, J_b = 4.4, 1 H), 3.90-3.99 (t, J = 9.8, 1 H), 4.12-4.19 (m, 1 H), 4.26-4.29 (d, *J* = 4.4, 1 H), 4.47-4.51 (q, 1 H), 5.65 (s, 1 H), 5.73-5.76 (d, *J* = 8.0, 1 H), 7.22-7.26 (d, *J*=8.0, 1 H), 9.48 (s, 1 H). ^{13}C NMR (400 MHz, $CDCl_3$) δ -4.9, -4.2, 18.4, 20.5, 22.9, 25.8, 26.0, 27.1, 27.4, 27.6, 67.7, 74.7, 75.5, 76.2, 94.1, 102.5, 139.5, 149.9, 163.5; HRMS (ESI$^+$) calcd for $C_{23}H_{42}N_2O_6Si_2$ $(M+H)^+$: 499.2660; found 499.2661.

1-((4a*R*,6*R*,7*R*,7a*R*)-2-(*tert*-butyl)-2-butyl-7-((*tert*-butyldimethylsilyl)oxy)tetrahydro-4*H*-furo[3,2-*d*][1,3,2]dioxasilin-6-yl)-3-((1-(6-nitrobenzo[*d*][1,3]dioxol-5-yl)ethoxy)methyl)pyrimidine-2,4(1*H*,3*H*)-dione (10). The silyl-protected uridine **9** (1.0g, 2.62 mmol) was dissolved in dry DMF (15 mL) and cooled to 0 ºC, and Cs_2CO_3 (2.0 g, 6.06 mmol) was added. NPOM chloride **3** (0.62 g, 2.38 mmol) was dissolved in DMF (1.5 mL), cooled to 0 ºC and the solution was slowly added into the protected uridine over 10 minutes and the reaction mixture was then allowed to warm to room temperature. After 16 hours, the solvent was removed by rotary evaporation and the residue was dissolved in EtOAc (100 mL) and water (50 mL), the organic layer was separated, washed with brine (3 x 15 mL), dried over anhydrous Na_2SO_4, filtered and evaporated. The oily residue was purified by column chromatography on silica gel using hexanes/EtOAc (6:1 to 3:1), delivering the product **10** as an off-white solid (1.12 g, 77%). ^{1}H NMR (400 MHz, $CDCl_3$):δ 0.12 (s, 3 H), 0.18 (s, 3 H), 0.91 (s, 9 H), 0.99-1.03 (m, 18 H), 1.45-1.48 (d, *J* = 6.4, 3 H), 3.75-3.84 (m, 1 H), 3.92-3.99 (m, 1 H), 4.05-4.16 (m, 1 H), 4.18-4.24 (dd, J_a = 16.4, J_b = 4.4, 1 H), 4.42-4.50 (m, 1 H), 5.13-5.21 (m, 1 H), 5.27-5.35 (m, 2 H), 5.54-5.59 (d, *J* = 18.4, 1 H), 5.64-5.67(dd, J_a = 8.0, J_b = 2.0, 1 H), 6.05-6.10 (m, 2 H), 7.11-7.14 (dd, J_a = 8.0, J_b = 3.2, 1 H), 7.16-7.20 (d, *J* = 11.2, 1 H), 7.40(s, 1 H). ^{13}C NMR (400 MHz, $CDCl_3$) δ -4.9, -4.2, -3.5, 18.3, 20.4, 22.8, 23.6, 23.7, 25.7, 25.9, 27.0, 27.5, 27.6, 67.6, 68.9, 69.5, 73.6, 74.6, 75.3, 75.4, 76.0, 94.3, 94.7, 101.7, 101.9, 102.9, 105.0, 106.5, 137.6, 137.8, 138.1, 147.0, 150.2, 152.3, 162.3; HRMS (ESI$^+$) calcd for $C_{33}H_{51}N_3O_{11}Si_2$ $(M+Na)^+$: 744.2960; found 744.2952.

1-((2*R*,3*R*,4*R*,5*R*)-3-((*tert*-butyldimethylsilyl)oxy)-4-hydroxy-5-(hydroxymethyl)tetrahydrofuran-2-yl)-3-((1-(6-nitrobenzo[*d*][1,3]dioxol-5-yl)ethoxy)methyl)pyrimidine-2,4(1*H*,3*H*)-dione (11). The silyl protected caged uridine **10** (157 mg, 0.22 mmol) was dissolved in DCM (0.9 mL) and cooled to 0 ºC. A chilled solution of hydrogen fluoride-pyridine (23.6 µL, 70% HF-pyridine, 0.91 mmol) in 145 µL pyridine was added and the reaction was stirred at 0 ºC for 1 h. The solution was washed with water (10 mL) and with a saturated aqueous $NaHCO_3$ (10 mL). The organic layer was dried with Na_2SO_4, filtered and concentrated via rotatory evaporation. The residue was purified by column chromatography on silica gel using $CHCl_3$/MeOH (100:1 to 25:1), furnishing the product **11** as an off-white solid (98 mg, 77%). ^{1}H NMR (400 MHz, $CDCl_3$): δ 0.07-0.08 (d, *J* = 2.8, 3 H), 0.09-0.10 (d, *J* = 6.0, 3 H), 0.87-0.88 (d, *J* = 1.6, 9 H), 1.44-1.47 (dd, J_a = 4.2, J_b = 2.0, 3 H), 3.79-3.83 (dt, J_a = 12.4, J_b = 3.0, 1 H), 3.92-4.00 (m, 1 H), 4.05-

4.09 (m, 1 H), 4.12-4.17 (dt, J_a = 12.4, J_b = 4.8, 1 H), 4.35-4.38 (q, 1 H), 5.11-5.12 (dd, J_a = 21.8, J_b = 9.8, 1 H), 5.27-5.33 (m, 2 H), 5.56-5.62 (dd, J_a = 16.0, J_b = 4.4, 1 H), 5.63-5.70 (dd, J_a = 21.8, J_b = 4.4, 1 H), 6.04-6.09 (m, 2 H), 7.14-7.20 (d, J = 14.8, 1 H), 7.40 (s, 1 H), 7.75-7.77 (d, J = 8.4, 1 H). ^{13}C NMR (400 MHz, $CDCl_3$) δ -5.2, -4.7, 18.0, 23.5, 23.7, 25.7, 61.3, 61.6, 68.9, 69.3, 70.0, 70.5, 72.8, 73.5, 75.1, 85.0, 85.1, 91.0, 92.1, 101.6, 101.9, 102.9, 103.0, 104.9, 106.4, 136.6, 137.5, 137.6, 140.4, 147.0, 149.4, 150.8, 152.2, 162.6; HRMS (ESI^+) calcd for $C_{25}H_{35}N_3O_{11}Si$ $(M+H)^+$: 604.1939; found 604.1953.

1-((2*R*,3*R*,4*R*,5*R*)-5-((bis(4-methoxyphenyl)(phenyl)methoxy)methyl)-3-((*tert*-butyldimethylsilyl)oxy)-4-hydroxytetrahydrofuran-2-yl)-3-((1-(6-nitrobenzo[*d*][1,3]dioxol-5-yl)ethoxy)methyl)pyrimidine-2,4(1*H*,3*H*)-dione (12). The uridine analog **11** (98 mg, 0.168 mmol) was dissolved in pyridine (2.5 mL), and 4,4'-dimethoxytrityl chloride (85.4 mg, 0.25 mmol) was added. The reaction mixture was then stirred at room temperature for 16 h. Methanol (1 mL) was added and the reaction was concentrated by rotatory evaporation. The residue was purified by column chromatography on silica gel using hexanes/EtOAc/Et_3N (6:1:0.25) then $CHCl_3$/MeOH/Et_3N (100:1:5) delivering the product **12** as a yellow solid. (126 mg, 85%). ^{1}H NMR (400 MHz, $CDCl_3$): δ -0.03-(-0.04) (d, J = 11.2, 3 H), 0.01-0.05 (d, J = 17.6, 3 H)0.74-0.75 (d, J = 1.6, 9 H), 1.30-1.33 (t, J = 6.0, 3 H), 3.30-3.33 (d, J = 6.0, 2 H), 3.60, (s, 6 H), 3.87-3.95 (m, 1 H), 4.04-4.16 (m, 2 H), 4.97-5.07 (m, 2 H), 5.12-5.22 (m, 2 H), 5.54-5.72 (m, 1 H), 5.81-5.83 (dd, J_a = 4.0, J_b = 1.2, 1 H), 5.84-5.89 (d, J = 22.0, 1 H), 6.65-6.67 (d, J = 8.4, 4 H), 7.03-7.19 (m, 9 H), 7.17-7.19 (m, 1 H), 7.24-7.26 (d, J = 9.2, 1 H), 7.64-7.75 (m, 1 H). ^{13}C NMR (400 MHz, $CDCl_3$) δ -5.2, -4.5, 14.2 18.1, 23.6, 23.8, 25.7, 55.3, 60.4, 61.5, 62.2, 69.0, 69.6, 70.2, 72.9, 73.9, 76.5, 83.1, 83.4, 87.1, 89.5, 89.7, 101.3, 101.6, 102.9, 104.8, 106.4, 113.3, 123.8, 127.2, 128.1, 130.1, 135.0, 135.2, 135.3, 136.0, 137.7, 138.0, 138.7, 142.0, 144.3, 146.9, 149.8, 150.6, 152.0, 152.2, 158.7, 162.3; HRMS (ESI^+) calcd for $C_{46}H_{53}N_3O_{13}Si$ $(M+Na)^+$ 906.3245, found 906.3606.

(2*R*,3*R*,4*R*,5*R*)-2-((bis(4-methoxyphenyl)(phenyl)methoxy)methyl)-4-((*tert*-butyldimethylsilyl)oxy)-5-(3-((1-(6-nitrobenzo[*d*][1,3]dioxol-5-yl)ethoxy)methyl)-2,4-dioxo-3,4-dihydropyrimidin-1(2*H*)-yl)tetrahydrofuran-3-yl (2-cyanoethyl) diisopropylphosphoramidite (13). The DMT protected caged uridine **11 (**0.24 g, 0.27 mmol) was dissolved in DCM (5 mL) and chilled to 0 °C and flushed with N_2 gas. DIPEA (0.14 g, 1.08 mmol) was added and the reaction mixture was stirred at 0 ºC for 10 min $NCCH_2CH_2OP[N(i\text{-}C_3H_7)_2]Cl$ (0.13 g, 0.54 mmol, 2 eq.) was added and the resulting reaction mixture was gradually allowed to warm to room temperature over 12 h. MeOH (0.5 mL) was added and the reaction was concentrated by rotatory evaporation. The residue was purified by column chromatography on silica gel using hexanes/$CHCl_3$/Et_3N (100:20:1), delivering the product **12** as a foamy yellow solid (0.26 g, 91%). ^{1}H NMR: (400 MHz, $(CD_3)_2CO$): δ 0.16-0.20 (m, 6 H), 0.93-0.97 (m, 9 H), 1.06-1.09 (dd, J_a = 6.8, J_b = 2.4, 3 H), 1.17-1.20 (m, 9 H), 1.46-1.47 (d, J = 6.4, 3 H), 2.43-2.50 (q, 1 H), 2.59-2.66 (q, 1 H), 2.76-2.82 (m, 1 H), 3.27-3.31 (m, 1 H), 3.44-3.76,(m, 6 H), 3.80-3.81 (t, J = 1.6, 6 H), 4.28-4.52 (m, 3 H), 5.13-5.20 (t, J = 6.0, 1 H), 5.22-5.36 (m, 2 H), 5.41-5.45 (m, 1 H), 5.76-5.95 (m, 1 H), 6.13-6.20 (m, 2 H), 6.91-7.13 (td, J_a = 8.8, J_b = 2.0, 4 H), 7.14-7.28 (dd, J_a = 15.8, J_b = 1.4, 1 H), 7.28-7.33 (m, 1 H), 7.32-7.39 (m, 6 H), 7.46-7.52 (m, 3 H), 7.76-7.88 (m, 1 H). ^{13}C NMR (400 MHz, $(CD_3)_2CO$) δ -4.3, -4.2, 14.7, 15.6, 18.8,

20.9, 23.9, 24.1, 24.4, 25.0, 25.2, 25.3, 26.4, 29.6, 29.8, 30.0, 30.2, 30.4, 43.8, 44.1, 47.1, 55.7, 58.9, 59.7, 60.7, 63.3, 63.5, 70.0, 70.2, 72.6, 73.1, 73.9, 74.4, 76.0, 76.6, 77.6, 83.3, 83.6, 90.3, 90.7, 100.5, 101.9, 104.3, 104.5, 105.2, 105.4, 106.9, 107.6, 114.2, 128.0, 128.9, 129.2, 131.2, 135.9, 136.2, 138.8, 139.5, 139.7, 142.7, 144.3, 145.6, 151.6, 153.2, 154.5, 159.9, 162.5; ^{31}P NMR (400 MHz, $(CD_3)_2CO$): δ = 147.6, 147.9, 148.1.
